# The Standard Model, the Maximalists and the Minimalists: New Interpretations of Trypillia Mega-Sites

**DOI:** 10.1007/s10963-017-9106-7

**Published:** 2017-09-02

**Authors:** John Chapman

**Affiliations:** Department of Archaeology, Durham University, South Road, Durham, DH1 3LE UK

**Keywords:** Low-density urbanism, Trypillia, Chalcolithic, Human impact, Settlement planning

## Abstract

The currently prevailing view of the Trypillia mega-sites of the fourth millennium BC has been the dominant model for over 40 years: they were extra-large settlement examples of the Childean ‘Neolithic package’ of permanent settlement, domesticated plants and animals, and artifact assemblages containing polished stone tools and pottery. Trypillia mega-sites have therefore been viewed as permanent, long-term settlements comprising many thousands of people. This view of these extraordinary sites has been identical whatever the various opinions on their urban or other status. In recent mega-site publications, a maximalist gloss has been put on this standard view—with population estimates as high as 46,000 people (Rassmann et al. in J Neolit Archaeol 16: 96–134, 2014). However, doubts about the standard view have been emerging over the past two decades. As a result of the last six years’ intensive investigations, a tipping point has been reached, with as many as nine lines of independent evidence combining to create such doubts that the only logical response is to replace the standard model (not to mention the maximalist model) with a version of the minimalist model that envisions a less permanent, more seasonal settlement mode, or a smaller permanent settlement involving coeval dwelling of far fewer people (the ‘middle way’). In this article, I seek to construct an evidential basis for the alternatives to the standard view of Trypillia mega-sites.

## Introduction: Eurasian Urbanism

As we have seen from the Introduction to this issue (Gaydarska 2017), the Eurasian urbanism debate is dominated by old evolutionary concepts: the Childean model and the Graeco-Roman high-density model. Sites considered ‘urban’ were usually large, high-density, with developed hinterlands, and boasted a proportion of the characteristics deemed ‘urban’ in a check-list. There is now massive tension between the old models and the new-found urban diversity in Eurasia, Africa and the New World. There are two approaches to this conundrum. First, the recognition of a major form of low-density urbanism, which in itself is regionally varied while sharing several important characteristics (cf. Fletcher 2009); and secondly, the identification of local, relational solutions rather than essentialist answers to the urban problem (Gaydarska 2016). What I seek to identify in this article is a form of settlement that came early in the trajectory of Eurasian urbanism, did not necessarily constitute an aspect of the traditional Childean urban model, and did not necessarily lead to the development of a further urban form. We now turn to the Trypillia mega-sites—a distinctive phenomenon forming part of the Cucuteni–Trypillia group.

## The Cucuteni–Trypillia Group

**Fig. 1:**
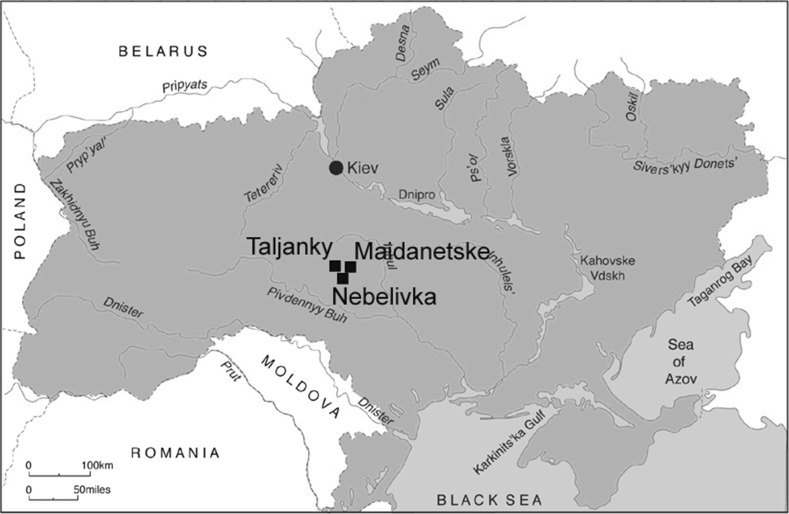
Location map of Cucuteni–Trypillia groups, showing the important Trypillia mega-sites of Taljanky, Nebelivka and Maidanetske. *Source* Marco Nebbia (2017)

The time–place distribution of the Cucuteni–Trypillia (or ‘CT’) group—two millennia (4800–2800 cal BC) and over 200,000 km^2^—makes it one of the largest and longest-lasting groups in ‘Old Europe’ (Fig. 1). Three key points stand out from the long history of CT studies: the strong predominance of the domestic domain over the mortuary sector in both the Cucuteni and the Trypillia group; the closely related near-absence of the materialization of hierarchies in either group; and the differential development of massive sites (the so-called ‘mega-sites’) in certain zones of the Trypillia group but not in others, and their complete absence from the Cucuteni sites (Videiko 2012; Monah and Monah 1997, pp. 21–34).

The Marxist philosopher, Slavoj Žižek (Slovenia) has elaborated the concept of the ‘Big Other’ (Žižek 2007)—an idea or set of ideas sufficiently general and significant to attract the support of most members of society yet, at the same time, sufficiently ambiguous to allow the kinds of localized alternative interpretations that avoid constant schismatic behavior. Sheila Kohring (2012) considers the Big Other to be a link between the structuring of a group’s symbolic world and its creation of material traditions. In CT, the materialization of the Big Other took place mainly through houses, figurines and painted pottery (Chapman and Gaydarska in press).CT houses manifested an entire worldview for their occupants, creating a warm, safe, comfortable, decorated, ritualised and monumental place, which was reproduced over an estimated 70 successive generations (Burdo et al. 2013). A common CT practice involved the deliberate burning of the house at the end of its use-life (Johnston et al. in press). Many thousands of fired clay anthropomorphic figurines are known from CT settlements (Monah 2012). Sets of complete figurines were used and deposited in structures thereby interpreted as shrines, while fragmentary figurines were deposited in houses, pits or the occupation level. Decorated pottery comprised both fine wares (painted in the western part, incised in the east) and coarse wares (mostly incised and/or impressed) (Tsvek and Rassamakin 2005). Pottery dominated the ‘grave goods’ deposited in mortuary house-burning ceremonies, with finely painted wares a prestige good in their own right. The house, the figurine and the painted vessel dominated the Trypillia Big Other, which stood in strong contrast to the minimal discard of metal objects, whether copper, silver or gold (a rare exception is the small gold hair-ornament found at Nebelivka in the 2012 season in the so-called ‘mega-structure’: see below). The paucity of Trypillia graves and hoards (at least, after the Karbuna hoard, dated to the A phase: Dergachev 1998) may partly explain this phenomenon.

## The Standard Model, the Maximalists and the Minimalists

The development that differentiated Trypillia communities from all the other groups that made up ‘Old Europe’ (Gimbutas 1982) was the creation of massive settlements, termed ‘mega-sites’—settlements covering more than 100 ha (Videiko 2012) or 150 ha (Müller et al. 2016), the biggest of which constitute the largest settlements in fourth millennium BC Eurasia. The appearance of these settlements did not correlate with the emergence of a state in any form. While there has been considerable disagreement among Ukrainian prehistorians as to the urban or non-urban status of mega-sites, the vast majority of prehistorians have supported the ‘standard position’ on mega-sites: that they were long-term, permanently occupied central places with many thousands of people occupying the site at the same time. (Exceptions include Petrenko 2009 and Tkachuk 2010, both of whom raised the possibility of less permanent occupation.) Few prehistorians have felt the need to support this view with evidence, let alone challenge it. A close examination of the standard model indicates five strands of evidence which could be used in its defence: the cultural background; settlement planning; dwelling-houses and assembly houses; food and drink; and the scale of pottery production.

In the background to the Cucuteni–Trypillia group discussed above, it is not only the CT ‘Big Other’ that provides long-term continuity over two millennia—there are forms of settlement planning that can possibly be traced back almost a millennium before the advent of Nebelivka. It can also be argued that subsistence practices showed considerable signs of continuity over 1500 years.

The most obvious argument in support of the standard position concerns the formal settlement planning of the mega-sites, which embodied three identifiable principles discovered in the first phase of mega-site research: (1) the creation of settlements with concentric circuits of houses facing at right angles to the circuit, (2) the consistent use of inner radial streets impinging, to a greater or lesser extent, on (3) an essentially empty central space. These principles have been claimed for Trypillia Phase A sites such as Bernashivka and Mogylna III (Videiko 2012), albeit in embryonic form. The greatly expanded settlements in Trypillia Phase BII embodied a more formal recapitulation of the ancestral form but with variations (e.g. the creation of multiple concentric circuits at Maidanetske: Müller and Videiko 2016). The growth of settlements to include c. 1500 houses at Nebelivka, between 1600 and 2000 houses at Taljanky (Rassmann et al. 2016, p. 32), and almost 3000 houses at Maidanetske (Müller and Videiko 2016, p. 72) betokens a large number of inhabitants, even if a fraction of the houses were inhabited coevally. These high population estimates imply hierarchical order to cope with logistical issues of supply and demand and the settlement of disputes. It is a fundamental aspect of the standard model that high populations and hierarchical social order were both involved and interacted with each other.

**Fig. 2:**
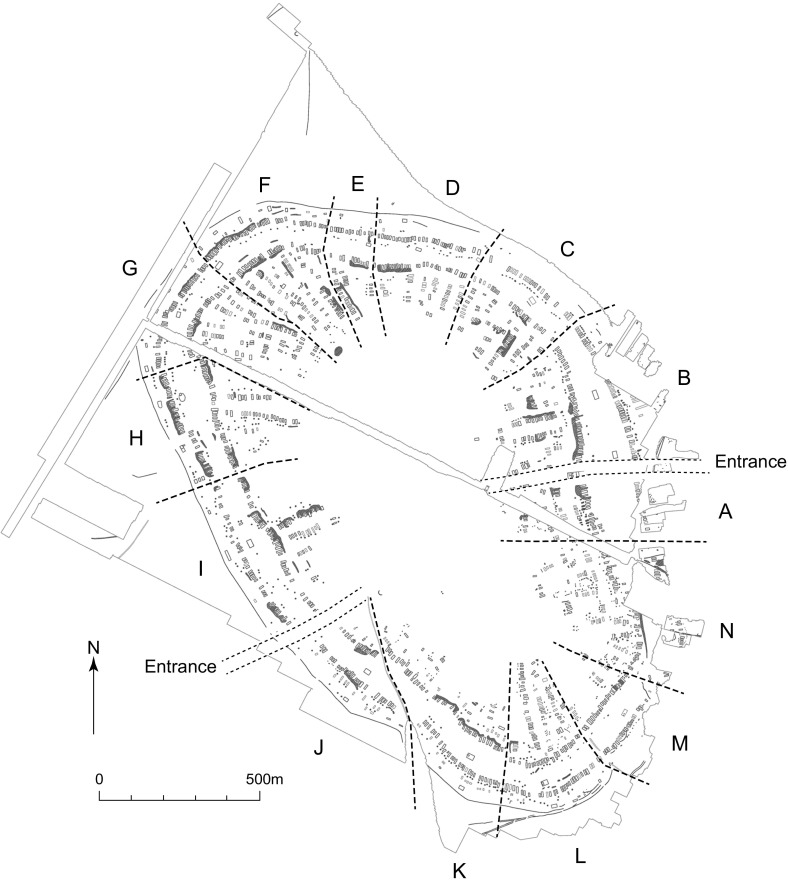
Geophysical plan of Nebelivka, with division into quarters. *Source* Y. Beadnell, based upon plan by Archaeological Services, Durham University

The coherence and regularity of mega-site plans suggest conscious decision-making in the implementation of ancestral planning principles (Fig. 2). Regularity is observed in the way that spatial divisions based, *inter alia*, upon the integration of assembly houses created communal units of broadly similar scale and number of neighbourhoods.

**Fig. 3:**
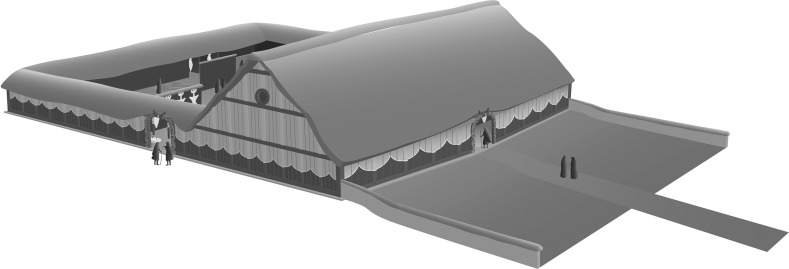
Reconstruction of the Nebelivka mega-structure. *Source* C. Unwin & S. Johnston

The construction of the dwelling houses as well as the assembly houses provides one of the strongest supports for the standard model. The houses typified solidity and permanence, creating a long-term investment in landscape monumentality, not least when the houses had two storeys. Both the building and the burning of the houses clearly took major organisation (Johnston et al. in press). The mega-structure (Chapman et al. 2014a) (Fig. 3) and the other assembly houses constituted prestige structures for an important centre but contained very few differentiated finds. However, taphonomic explanations for this absence include the scenario of participants in assembly house rituals taking their figurines home for secondary domestic use and deposition.

The production of food and drink for a large population depended upon traditional mixed farming practices developed in permanent settlements over 1500 years. Farming productivity relied on the high fertility of the chernozem.

Pashkevich’s (2005) summary charts for grain impressions in pottery and house daub present data combined from all sites of each of the three main phases. While the same plants were cultivated throughout the Trypillia period, these summary data indicate a long-term decline in wheat and hulled barley cultivation, with a major late increase in broomcorn millet. However, the low incidence in all phases of more productive bread wheats and naked barley suggests no obvious intensification of farming over time.

**Fig. 4:**
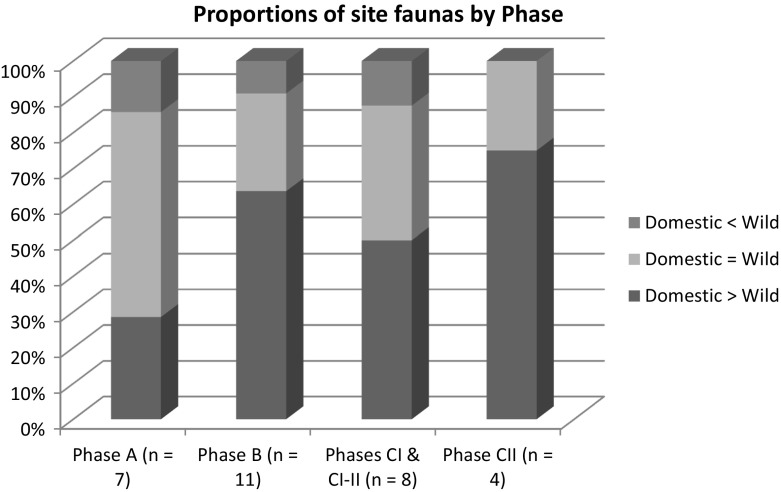
Proportions of site faunal assemblages by phase. Data from Zhuravlyov (2008)

The archaeozoological data are mixed and present serious issues of sample size: recent excavations at Nebelivka used flotation and sieving to produce a sample of over 2500 bone fragments, whereas most of the sites studied have yielded fewer than 1000 bone fragments (Zhuravlyov 2008). Unfortunately, no ‘taphonomic hygiene’ has been applied in the most recent synthesis of archaeozoological data (Kirleis and Dal Corso 2016). This leads to some rather general comments on the trends in animal preferences through the Trypillia period (Fig. 4) (sites are considered to have broadly similar numbers of wild and domestic animals within a 10% threshold, while a 20% difference is used to separate sites preferring wild over domestic or the converse). The principal difference was between sites in Phases A–CI and those dated to Phase CII. The only phase when wild animals were never preferred to domesticates was CII—when consumption of lamb and mutton was more frequent than before. This may also have been related to increasing interactions with steppe pastoralists in the North Pontic area, or the growing significance of woollen textiles.

Prior to Phase CII, there was a long-term fluctuation in the number of sites with a preference for domestic over wild animals—stronger in Phase B than in A or CI. But the clear preference on most of these pre-CII sites was for beef over pork. One possible diachronic trend was the growing proportion of sites with the strongest emphasis on domestic stock, viz. >80% of bone numbers. However, we should not overlook the finding of one site with an enhanced preference for wild game in each pre-CII phase. That we can make only such general comments after a century of excavations on Trypillia sites means that there is an urgent need for high-quality archaeozoological data from the recent fieldwork in Ukraine.

In summary, while both aspects of mixed farming could have underpinned increases in scale of dwelling, there is currently little evidence that either extensification or intensification of basic subsistence strategies occurred. Instead, we have a picture of rather traditional subsistence practices, with long-term preferences for similar plants and animals with regional variations in selected diets.

Other aspects of production also connect to the scale of practices. The sheer mass of artifacts—often pottery made from local clays—is consistent with large populations: the estimated 50–100 vessels in each burnt house assemblage require careful planning and site-wide organization. Although there is no evidence for pottery kilns at Nebelivka, such features have been excavated at Maidanetske and Taljanky (Korvin-Piotrovskiy et al. 2016), which implies large-scale consumption. Yet even the maximalists accept that the distribution of kilns at Maidanetske and Taljanky indicates dispersed rather than centralised ceramic production (Müller and Pollock 2016).

This summarises the case for the standard view of mega-sites as large, permanent aggregations of people. A gloss on the standard view is the maximalist position, in which population estimates range from 7500 to 46,000. (It may be noted in parenthesis that there were only 18 European cities with populations of 40,000 people c. AD 1500 Osborne 2007, p. 81).

## The Tipping Point

We have reached a tipping point with our recent investigations of Trypillia mega-sites, with too many contradictions to allow us to maintain the standard or maximalist views. Nine lines of argument can now be marshalled in favour of two more modest alternative interpretations, viz. the minimalist view of mega-sites as seasonal aggregation sites with much lower populations and a ‘middle way’, with much smaller populations living year-round at the mega-site.

### Mega-Site Planning and the Evidence of Quarters and Neighbourhoods

While it is true that the earlier mega-site plans shared coherence and regularity, a closer examination of the recent plans shows much more variability than once identified. In addition to the two extensive multi-functional ‘empty’ areas—an Inner Zone inside the inner radial streets and a Middle Zone between the main house circuits—the plans of Nebelivka and Maidanetske show an Outer Zone between the outer house circuit and the enclosing perimeter ditch (Chapman et al. 2014b) (Fig. 2). What is important is the lack of regularity in the layout of these zones at Nebelivka. The maximum width of the Middle Zone varied from 60 m to 140 m, while that of the Outer Zone varied from 40 m to 75 m. There is much variability in the way that design concepts are worked out locally, suggesting a bottom-up approach to planning.

**Fig. 5:**
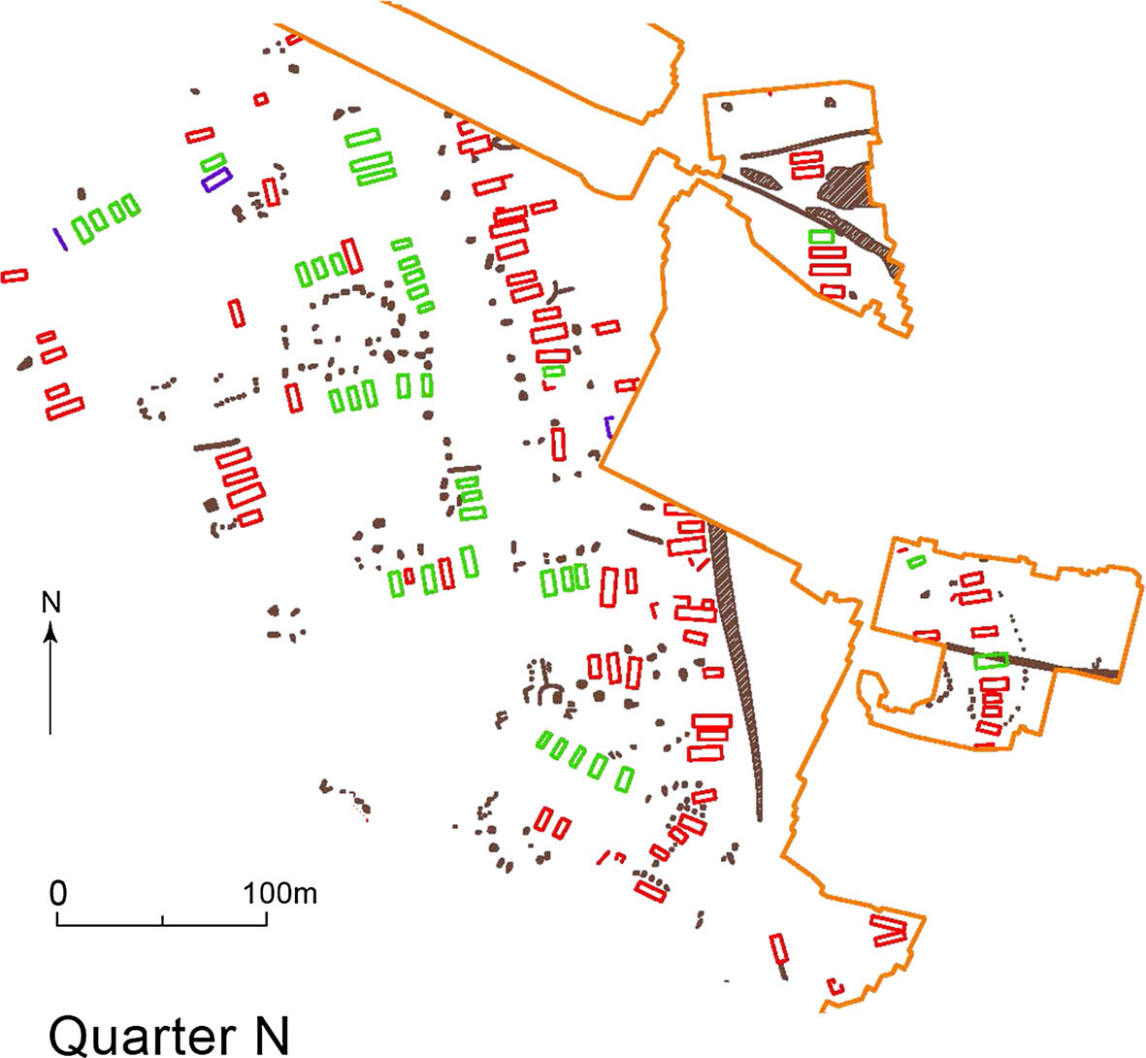
Quarter N, which included ‘Nebelivka Square’. *Source* Y. Beadnell, based upon plan by Archaeological Services, Durham University

This is also true for the neighbourhood and the quarter (Fig. 5) (and for Maidanetske and Taljanky: limitations of space do not permit a detailed analysis of these two plans). The number of houses in a neighbourhood ranged from 3 to 25 (Quarter L). While the majority of neighbourhoods consisted of linear arrangements of houses, there were several examples of house groups laid out around a central space resembling the plan of a founder settlement (e.g. the so-called ‘Nebelivka Square’: Fig. 4). There was also great variation in the number of pits in any given quarter, with totals ranging from 0 to 217 (Quarter N).

Quarters developed in such markedly different ways that no single quarter resembled any other in size or content. Quarters varied in width from 190 m to 560 m, with the number of neighbourhoods varying from 5 to 18. This again shows that the mega-site developed from the bottom up within the overall constraints of key ancestral planning principles.

In the standard view of mega-site development (e.g. Videiko 1996, fig. 6), the beginning of the Maidanetske mega-site occupation consisted of a small group of houses dispersed across the mega-site (Videiko’s Phase 1). There was no explanation of how the scattered houses related to overall mega-site planning principles and no model for how the house circuits developed from these early neighbourhoods into the consolidated house circuit of Phase 2. In other words, the problem of deriving complete house circuits from a limited initial mega-site occupation is not restricted to the minimalist view but is clearly shared by the maximalist model as well.

**Fig. 6:**
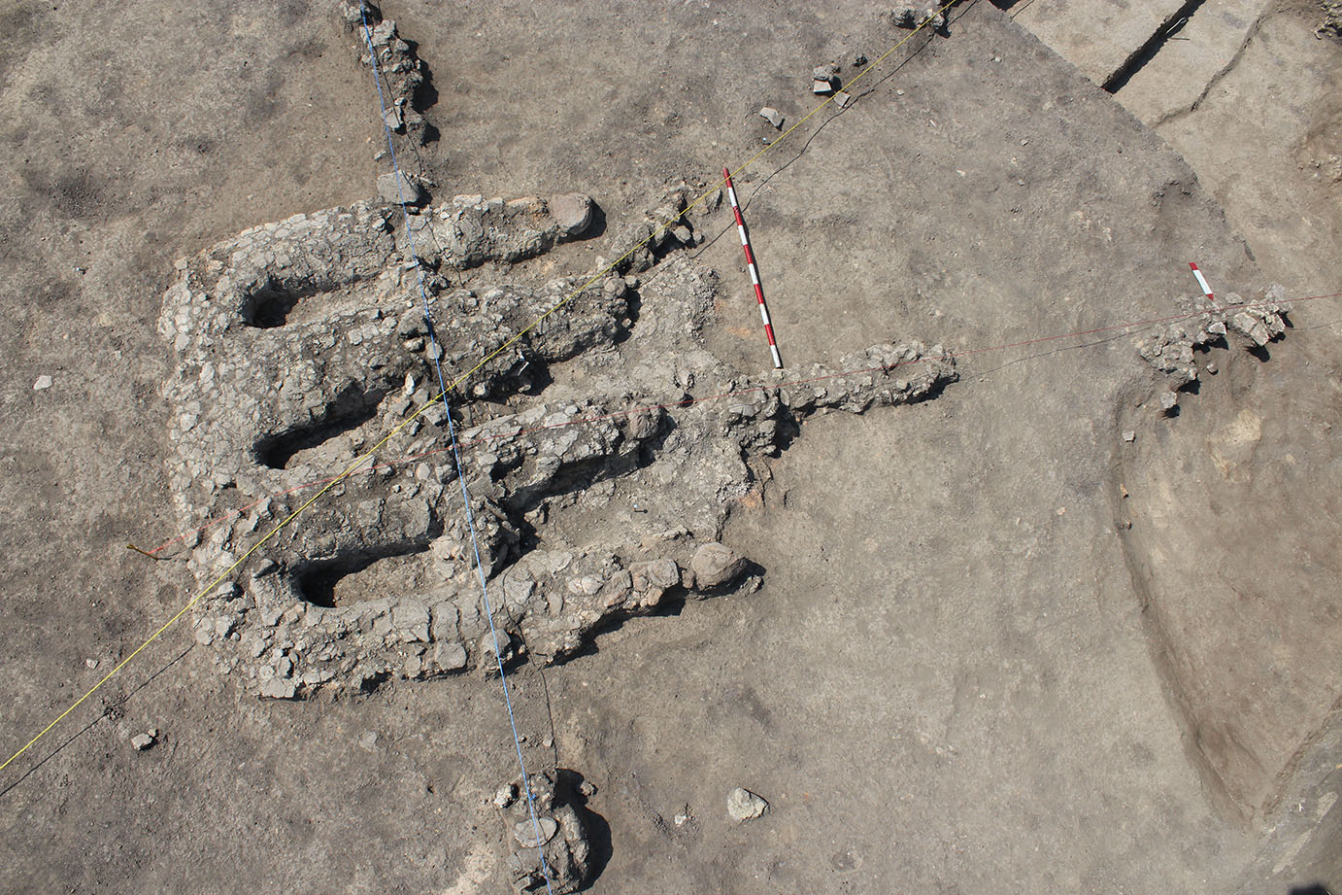
Communal cooking feature from above, Nebelivka. Photo: M. Videiko

### Assembly Houses and the Mega-Structure

If ‘local’ variability is the key to the growth of quarters and neighbourhoods, similar variability is found in the frequency, size, location and history of the larger structures we term ‘assembly houses’ because of their integrating functions within each quarter. The number of assembly houses in a quarter varied from none to three, with different residents probably building their own assembly house in sequence as a quarter developed over time. There was great variability in the spacing of assembly houses (from 100 m to over 800 m) and in their sizes—mostly within the range 120–475 m^2^, but with the largest covering an extraordinary 1320 m^2^, making it one of the largest structures in prehistoric Europe. Equally, the way that assembly houses were burned down varied across the mega-site, with a majority burnt round the walls and only a few completely burnt. While the assembly houses integrated different quarters, their form, location and mode of burning again suggest a bottom up approach to planning inconsistent with the standard view.

Although the largest assembly house (the ‘mega-structure’) was special in its sheer size (Chapman et al. 2014a) (Fig. 3), it resembled the standard Trypillia house in both its internal features (podium, platforms, bin) and the deposition of modest numbers of ceramics and figurines. The paucity of its special finds (limited to a set of 21 miniature vessels, a group of scattered fired clay tokens, and a single gold hair-ornament) gives no indication of the materialization of hierarchy. More figurines have been found in each of the excavated pits at Nebelivka than were found deposited in the mega-structure. The finding that the mega-structure was simply the household writ large raises questions about the supposed intra-site hierarchy, which could have operated at the level of the quarter or, more likely, the neighbourhood, with considerable ‘local’ autonomy of cultural practices within the mega-site overall.

### Scalar Stress

Johnson’s (1982) paper on ‘scalar stress’ dealt with the decision-making consequences of increasing numbers, using business studies and industrial psychological research to indicate that the key number seven was the maximum for face-to-face consensus-based decision-making and that more units required a higher-level co-ordinating group. For the mega-sites, the coeval occupation of 500 houses would have resulted in a four-level decision-making hierarchy, with household leaders represented in neighbourhood committees, neighbourhood committee leaders giving their views at quarter committees, and the final say left to the mega-site leader. This seems utterly improbable in the Neolithic—not least in the absence of any materialization of such a hierarchy. If the scalar stress approach was applied to the maximalists’ population estimates, this would imply a five-level hierarchy, with a governing body at the pinnacle. Such four- or five-level hierarchies do not easily fit into current views of European prehistory. Instead, a far smaller population organised through local neighbourhoods with some co-ordination of communal feasting and other events at the quarter level would have minimised the problems of scalar stress raised by the maximalists’ view, although issues of disputes between heterarchically-organised neighbourhoods can never be dismissed.

### Communal Cooking for Feasts

Much has been made of the impressive feature excavated at Nebelivka in 2014 (Fig. 6). The Ukrainian side interprets this as a pottery kiln, emphasizing the Childean urban criterion of craft specialization. However, since the modus operandi of this high-temperature feature is unclear, we suggest that it was not a kiln but a large-scale cooking facility to support feasting in the quarter or the neighbourhood. The geophysical investigations at Nebelivka identified no anomalies of such intensity as to be consistent with the kilns found at Maidanetske and Taljanky; at the two last-named sites, the presence of neighbourhood-level pottery production was not consistent with highly centralised ceramic production, as acknowledged by Müller and Pollock (2016). At Nebelivka, a large-scale cooking facility was consistent with the medium-way interpretation of a smaller population as much as with seasonal congregations. The disposal of large quantities of animal bones in the large pits adjoining houses at Nebelivka is again consistent with neighbourhood-centred feasting rather than massive quarter-wide consumption practices, complementing the special placed deposits typical of these pits.

### The Nebelivka Micro-Region: The Question of a Hinterland

Most urban theorists have assumed that the city and its hinterland of smaller sites were inter-dependent, bringing each other into being (Smith 2007). Urban sites entailed such high levels of resource provisioning that the contributions of the smaller sites are generally considered essential to social reproduction.

It is therefore noteworthy that the Anglo-Ukrainian Project’s intensive, systematic fieldwalking within a 5 km radius of Nebelivka yielded no sign of manuring scatters and no small sites at all (Nebbia 2017). The targeted fieldwalking of areas of high settlement potential within a 20 km radius produced a few small, contemporary Trypillia sites (Nebbia 2017). In the case of other mega-sites, the two small Trypillia sites of Mosurov 2 and 3 lay within 4.2 km of Taljanky, while the small sites of Talnoe 2 and 3 lay within 3.3 km of Maidanetske (Gaydarska 2003, Fig. 1). However, we cannot be certain that the occupation of these four small sites was coeval with that of the mega-sites. The results give no sense of rural hinterland at all for mega-sites, with all of the negative logistical connotations (e.g. the procurement of cereals or salt) representing a serious challenge to claims of classic urban–rural structure for Nebelivka.

### Extensive and Intensive Agriculture

There has long been a tension between the supposed size of mega-site populations and Pashkevich’s (2005) view that Trypillian agriculture was inefficient, based upon low-yield cereals and extensive in scale, relying on the natural fertility of the chernozem soils rather than additional agrarian practices. However, recent flotation results at Nebelivka and Maidanetske confirm the predominance of low-yield cereals. Addressing the population–intensification relationship, Bogaard (2014) suggests a general trend in Eurasian prehistoric farming: extensification was a more typical response to population growth than intensification, although ‘local’ intensification may also have developed near an expanding site (e.g. in the large open areas at the heart of the mega-sites).The evidence for plough agriculture is limited to two plough models of dubious provenance (Ciuk 2008), while the Nebelivka fieldwalking showed no evidence for manuring practices. Equally, the lack of hinterland sites cannot support the extensification of Trypillian agriculture, which in turn would have relied upon the current evidence for wheeled transport and sledges (Ohlrau et al. 2016, p. 209). In the absence of either intensification or extensification of mega-site agriculture, Pashkevich’s model for Trypillian agriculture may be accepted as the appropriate, sustainable level for Trypillia cultivation. This kind of farming, however, would have been unable to support the high population levels of the maximalist model; rather, the new archaeobotanical results indicate much lower population levels or seasonal settlement.

### The Provision of Salt

It is now generally accepted that salt was one of the key resources for all prehistoric populations (Monah et al. 2007). Previous modelling of salt demand for CT sites (Chapman and Gaydarska 2003) has been applied to differing population estimates for Maidanetske (Table 1). For the lowest population estimate of 7500 people, salt procurement is required on a large scale, while an unimaginably vast scale was required for the highest population estimates (we have to accept that these figures would defeat even the mighty power of cattle-drawn sledges). The current state of prehistoric salt exploitation has yet to resolve a paradox: the undeniable evidence for Cucuteni salt exploitation in the Eastern Carpathian piedmont zone—with complex transportation demands across several major interfluves—can be contrasted with the logistically easier movement of salt from the Black Sea limans up the Southern Bug to the mega-site heartlands but the as yet complete absence of salt production sites on the Black Sea littoral (Mircea and Alexianu 2007). The alternative of a seasonal mega-site population, with visitors to festivals or pilgrimages bringing their own salt, or a small population reliant on other visiting groups for provisioning the mega-site, presents a more achievable model for salt provisioning.

**Table 1:** Estimated annual salt requirements for the various population estimates for Maidanetske^a^ (pp. numbers from Müller et al. 2016), based upon the basic neighbourhood module of 100 people, 30 cattle and 150 sheep (*source*: Chapman and Gaydarska 2003)

Population estimate	Low (kg)	Medium (kg)	Elite (kg)	High (kg)
7500 (p. 133)	33,750	63,450	101,250	79,650
11,000 (p. 207)	49,500	93,060	148,500	116,820
14,100 (p. 44)	63,450	119,286	190,350	149,742
15,000 (p. 133)	67,500	126,900	202,500	159,300
25,000 (p. 133)	112,500	211,500	337,500	265,500

^a^In this Table, we have omitted the highest population estimate for Maidanetske of 46,000 people (Rassmann et al. 2014) on the grounds that, in the 2016 volume, the ‘maximalists’ appear to have withdrawn from this extreme position

### The House-Building and Burning Experiment

In 2014, the Anglo-Ukrainian Project constructed 2/3 scale imitations of Trypillia houses—one one-storey and the other two-storey, with the aim of studying house construction and the results of house-burning. For safety reasons, only the two-storey house was burnt down in May 2015 (Johnston et al. in press). This experiment confirmed the surprising scale of resource use for both building and burning houses. While a total of 3 m^3^ of good-quality timber was required for the construction of the two-storey house, close to ten times more was needed for the successful burning of this house. This means that the construction of a module of 100 standard 12.7 m × 4.8 m. houses would have left a major deforestation signal in the pollen diagram. Moreover, burning this same module of 100 houses would have required the collection and combustion of 2 million ‘Nebelivka standard’ trees (defined as 2 m in height with a diameter of 20 cm), producing a major fire event in the pollen record. This estimate would rise to 20 million trees for the burning of over a thousand houses at the end of the Nebelivka occupation proposed by the maximalists.

### The Nebelivka Pollen Core

Sediments dating to the Middle Holocene (before, during and after the mega-site occupation) were located and cored at <300 m from the northeastern edge of the Nebelivka mega-site (Albert et al. submitted). Our expectation was that the mega-site would have produced a massive human impact signal, but the scale of the human impacts was far less than expected throughout the diagram. While it can be objected that the Nebelivka 1B diagram cannot yet be dated to a decadal level, the key point is that wherever the occupation of the mega-site is placed relative to the pollen core, there are no instances of strong human impact anywhere in the diagram. In summary, there were five unexpected absences: (1) deforestation episodes or sustained forest clearance (in fact, one re-afforestation episode occurred in the mega-site period); (2) big cereal peaks (a low cereal curve was present); (3) big pastoral peaks; (4) major charcoal peaks (nine separate ‘fire events’ were defined, with only two dating to the mega-site period); and (5) big erosion with high sedimentation rates (the slowest sedimentation rates occurred in the mega-site period). These absences are hard to explain in the context of a large, full-time, permanent occupation, whether in relation to house-building and house-burning phases or the intensification and/or extensification of agriculture and pastoralism. The Nebelivka 1B core provides the greatest challenge to the standard and maximalist notions of mega-site dwelling. The most obvious interpretation of the findings on human impact is a far smaller population creating and burning far fewer houses, cultivating far fewer crops and tending far fewer animals than is postulated in the standard account. What the diagram supports is an alternative model based upon three premises: a low-intensity arable model, consistent with Pashkevich’s model; a long-term pattern of small-scale house burning, with a small number of house-burnings every year and the occasional combustion of a larger number of houses to produce the twin peaks in large-size charcoal; and no obvious evidence for a maximalist occupation of all of the houses in the last phase of settlement at Nebelivka.

### Discussion: The Transformation of Identity

These findings show that the standard and the maximalist views of Trypillia mega-sites are no longer tenable. The two alternatives are: (1) the minimalist view of a seasonal occupation of a much smaller population in some form of congregation site (perhaps as a summer aggregation or a pilgrimage site); and (2) a ‘middle way’, in which much smaller populations than currently envisaged in the standard models dwelt at the mega-sites on a year-round basis.

In the minimalist view, mega-sites are seasonal agglomeration centres, with the active participation of hundreds of pilgrims or festival-goers from many smaller sites. There are two key components of the mega-site: (1) a small permanent population maintaining the central site, contributing their own subsistence labour for out-of-season dwelling; and (2) the vast majority of visitors to the mega-sites coming from up to 100 km away, from many small settlements, and bringing their own pottery, figurines, food and salt, building their own houses and burning them after several visits. The mega-sites were congregation sites, hosting the central gatherings of the Trypillia annual calendar.

The second model is the ‘middle way’, in which a much smaller permanent, year-round, long-term population is proposed for the mega-sites. In this view, the mega-sites remained central places in a relatively empty landscape. This model takes into account the evidence for coherence and consistency of planning at all the mega-sites and the presence of neighbourhood pottery production. No detailed modeling of the middle way has yet taken place but the initial estimate of population would be in the order of two or three thousand people who moved to Nebelivka from no more than sixty small settlements. The next stage of research is to model the two more modest views of Trypillia mega-sites and compare the results with the nine lines of evidence summarised here.

What does this mean for the status of mega-sites on the urban–non-urban continuum? Whichever of the four models of mega-sites is accepted, it is clear that mega-sites were low-density settlements constructed within an ancestral framework of specific, readily expandable planning principles, whether they were hierarchically structured, heterarchical or operated in a more egalitarian fashion. The standard and the maximalist views were based upon the dramatic size of population consonant with urban forms, as well as a necessary intensification in resource procurement—whether cereals, meat, flint or salt. By contrast, the middle way and the seasonal models shed the argument of population size as an urban indicator (Cowgill 2004) while maintaining their vast size. Their size and complexity remain important arguments for a different kind of urbanism. The implication of the seasonal and middle-way views is the presence of far fewer people more widely dispersed over the mega-site, thus lowering an already low settlement density. In the introduction to this Special Issue, Gaydarska (2017) has proposed two variables which help to distinguish urban from non-urban settlements: centrality and intensification. The paucity of small sites within 20 km of the mega-sites raises the question ‘to what were mega-sites central?’ A realistic answer is obtained by looking at a radius of 50 km or 100 km—distances which pilgrims or visitors to seasonal festivals could well have travelled within a week from the clusters of small sites identified by Nebbia (2017).

Given the uncertainty over intensification of agriculture, and having no statistics to compare the intensity of pottery and figurine discard on sites of varying sizes, I propose that the principal form of intensification focused on the third element of the Big Other—the houses. In the mega-sites, the traditional house was expanded to form sets of long-houses, with the establishment of assembly houses at some of the mega-sites culminating in the building of the Nebelivka mega-structure. The sheer number of houses built at Nebelivka was the third form of house-based intensification. It was the focus on intensification of the house, rather than public temples with their figurines, or prestige metal objects, that gave a distinctive character to the Trypillia mega-sites—one of the few groups of monumental sites which did not make use of stone monuments.

## Conclusions

The standard view of mega-sites as long-term, year-round dwelling places for thousands of people has dominated Trypillia archaeology for the last 40 years. It is not surprising that the largest settlements in fourth millennium BC Europe have been interpreted in such terms: after all, the planning principles and the number of large, well-built houses fitted the idea of a hierarchical society with skills in top-down planning. However, we have reached a tipping point where we can finally challenge the standard and maximalist views. We propose two alternatives: the middle way of a smaller but still permanent, year-round settlement; and the minimalist view of a seasonal congregation site with small groups in permanent occupation.

In the guise of smaller centres or seasonal agglomeration sites, the Trypillia mega-sites have something new to offer to the understanding of urban developments. The seasonal agglomeration sites may be regarded as low-density, egalitarian, central sites, while the middle-way mega-sites exceeded the possibility of the ‘large village’ concept, their highly structured central form being more suggestive of a heterarchical order based upon neighbourhoods of comparable but contrasting identities—a low-density heterarchical urban centre. Further modeling is required at Nebelivka to see the extent to which each of these competing notions can match the Bayesian modeling of the AMS dates.
